# ATF3 controls proliferation of osteoclast precursor and bone remodeling

**DOI:** 10.1038/srep30918

**Published:** 2016-08-02

**Authors:** Kazuya Fukasawa, Gyujin Park, Takashi Iezaki, Tetsuhiro Horie, Takashi Kanayama, Kakeru Ozaki, Yuki Onishi, Yoshifumi Takahata, Yukio Yoneda, Takeshi Takarada, Shigetaka Kitajima, Jean Vacher, Eiichi Hinoi

**Affiliations:** 1 Laboratory of Molecular Pharmacology, Division of Pharmaceutical Sciences, Kanazawa University Graduate School, Kanazawa, Ishikawa 920-1192, Japan; 2 Department of Biochemical Genetics, Medical Research Institute, Tokyo Medical and Dental University, Tokyo 113-8510, Japan; 3 Institut de Recherches Cliniques de Montréal (IRCM), Département de Médecine, Université de Montréal, Montréal, Québec H2W 1R7, Canada

## Abstract

Bone homeostasis is maintained by the sophisticated coupled actions of bone-resorbing osteoclasts and bone-forming osteoblasts. Here we identify activating transcription factor 3 (ATF3) as a pivotal transcription factor for the regulation of bone resorption and bone remodeling under a pathological condition through modulating the proliferation of osteoclast precursors. The osteoclast precursor-specific deletion of ATF3 in mice led to the prevention of receptor activator of nuclear factor-κB (RANK) ligand (RANKL)-induced bone resorption and bone loss, although neither bone volume nor osteoclastic parameter were markedly altered in these knockout mice under the physiological condition. RANKL-dependent osteoclastogenesis was impaired *in vitro* in ATF3-deleted bone marrow macrophages (BMM). Mechanistically, the deficiency of ATF3 impaired the RANKL-induced transient increase in cell proliferation of osteoclast precursors in bone marrow *in vivo* as well as of BMM *in vitro*. Moreover, ATF3 regulated cyclin D1 mRNA expression though modulating activator protein-1-dependent transcription in the osteoclast precursor, and the introduction of cyclin D1 significantly rescued the impairment of osteoclastogenesis in ATF3-deleted BMM. Therefore, these findings suggest that ATF3 could have a pivotal role in osteoclastogenesis and bone homeostasis though modulating cell proliferation under pathological conditions, thereby providing a target for bone diseases.

The integrity of the skeleton as well as bone modeling and remodeling is believed to be coordinately regulated by two different types of cells, i.e., bone-forming osteoblasts and bone-resorbing osteoclasts^1^,^2^. An imbalance of the sophisticated regulation between osteoclasts and osteoblasts leads to pathogenesis as well as the etiology of certain metabolic bone diseases including osteoporosis or osteopetrosis^3^. Several transcription factors have been identified as pivotal regulators of cell differentiation and function of osteoblasts and osteoclasts during skeletogenesis and bone remodeling^4^. Runx2 is a cell-specific member of the Runt family of transcription factors, which plays a critical role in cellular commitment/differentiation processes in osteoblasts^5^,^6^,^7^,^8^. Osterix is a zinc finger-containing transcription factor specifically expressed by osteoblasts of all skeletal elements to promote osteoblast differentiation^9^. In contrast, nuclear factor of activated T cells cytoplasmic 1 (NFATc1), which is specifically and potently induced by receptor activator of nuclear factor-κB (RANK) ligand (RANKL), is well-known as the key transcription factor of osteoclastogenesis^10^ in spite of its pivotal role in osteoblast function^11^.

The transcription factor activating transcription factor 3 (ATF3) belongs to the ATF/cAMP-responsive element-binding protein (CREB) family and regulates mRNA expression of various genes though its binding to the ATF/CREB *cis*-regulatory element^12^,^13^. Among the ATF/CREB family, ATF4 expressed by osteoblasts is shown to be a positive regulator of both osteoblastogenesis and osteoclastogenesis^14^. Moreover, CREB also has a pivotal role in osteoblast proliferation and differentiation^15^. ATF3 has been shown to be implicated in the pathogenesis of various diseases, including metabolic diseases, immune diseases, inflammatory diseases, or cancer, as well as the regulation of cell proliferation and differentiation by regulating patterns of gene expression in various types of cells^16^,^17^,^18^,^19^. We have recently demonstrated that ATF3 regulates the development of experimental osteoarthritis without affecting normal chondrogenesis under pathological conditions through its expression in chondrocytes^20^. ATF3 is identified as a negative regulator of osteoblast differentiation by an *in vitro* study^21^. Although ATF3 has a critical role in cell function of neutrophil and macrophage among mature myeloid cells^22^,^23^,^24^, little attention has been paid to its implication in the pathogenesis of bone diseases as well as in the regulation of osteoclastogenesis till date.

Expression profile of the ATF family during osteoblastogenesis and osteoclastogenesis *in vitro* revealed that *ATF3* expression is highest at precursor stages of both osteoclasts and osteoblasts. To establish the pivotal role of ATF3 in bone homeostasis, we generated cell-specific (osteoblast-specific and osteoclast precursor-specific) *ATF3* deficient mice. In this study, we showed the crucial role of ATF3 in the cell-autonomous regulation of osteoclastogenesis and bone resorption *in vitro* and *in vivo*. Subsequent analyses identified that ATF3 regulates RANKL-induced cell proliferation in osteoclast precursors. In addition, we found that ATF3 regulates cyclin D1 expression though modulating activator protein-1 (AP-1)-dependent transcription in osteoclast precursor. Our results demonstrated that the RANK-ATF3-cyclin D1 axis in osteoclast precursors is an essential pathway for the regulation of osteoclastogenesis and bone remodeling.

## Results

### Conditional deletion of ATF3 in osteoclast precursors protects RANKL-induced osteoclast activation and bone loss

Among different ATF family members, expression of *ATF1* and *ATF4* was gradually decreased during osteoclastogenesis *in vitro*, with constitutive constant expression of *ATF2* and *ATF6*. Conversely, expression of *ATF3* and *ATF5* was highest in undifferentiated osteoclasts (day 0), and their expression levels were constantly very low during osteoclast differentiation (day 1–day 4) (Supplemental Fig. S1). Moreover, expression of *ATF3* was also highest in undifferentiated osteoblasts *in vitro*, and its expression was constitutively low during osteoblast differentiation (day 7–day 28) (Supplemental Fig. S2). These results suggest that ATF3 has a pivotal role in cellular function at precursor stages of both osteoclasts and osteoblasts.

**Figure 1:**
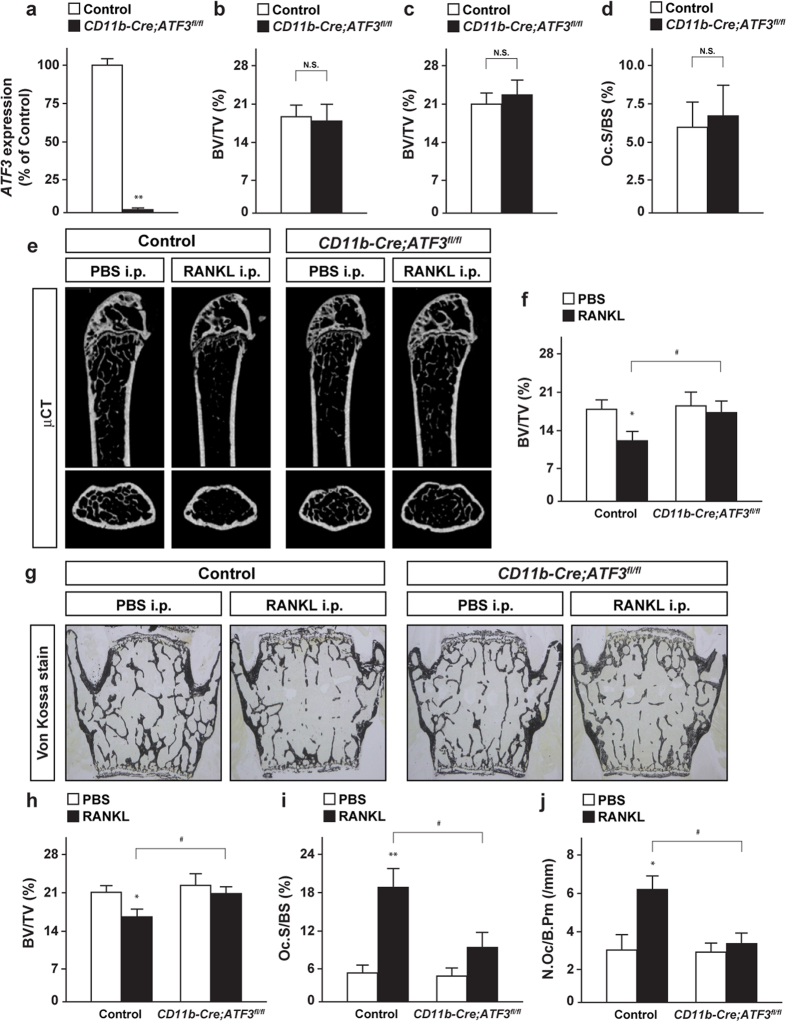
ATF3 expressed by osteoclast precursors is implicated in RANKL-induced bone loss. (**a**) *ATF3* expression in CD11b positive cells in bone marrow of control and *CD11b*-*Cre;ATF3*^*fl/fl*^ male mice (*n* = 4). (**b**) BV/TV of femurs, and (**c**) BV/TV and (**d**) Oc.S/BS of vertebrae of control and *CD11b*-*Cre;ATF3*^*fl/fl*^ male mice at 12 week-old (control, *n* = 7; *CD11b*-*Cre;ATF3*^*fl/fl*^, *n* = 9). (**e**) μCT analysis and (**f**) BV/TV of femurs, and (**g**) Von Kossa staining, (**h**) BV/TV, (i) Oc.S/BS and (**j**) N.Oc/B.Pm of RANKL-injected mice (control-PBS, *n* = 7; control-RANKL, *n* = 8; *CD11b*-*Cre;ATF3*^*fl/fl*^-PBS, *n* = 6; *CD11b*-*Cre;ATF3*^*fl/fl*^-RANKL, *n* = 9). *P < 0.05, **P < 0.01, significantly different from the value obtained for (**a**) control mice or (**f,h–j**) PBS-injected mice. ^#^P < 0.05, significantly different from the value obtained for RANKL-injected control mice.

To evaluate the physiological importance of ATF3 in bone homeostasis *in vivo*, we first generated osteoclast precursor-specific ATF3 knockout mice by crossing ATF3-floxed mice with *CD11b-Cre* mice. Mice lacking ATF3 in CD11b-expressing cells, hereafter referred to as *CD11b-Cre;ATF3*^*fl/fl*^ mice, were normal when compared with control mice in terms of physical appearance, fertility, body weight, and naso-anal length (data not shown). ATF3 expression was markedly abolished in CD11b positive cells in the bone marrow of *CD11b-Cre;ATF3*^*fl/fl*^ mice (Fig. 1a), in accordance with a marked Cre-mediated excision at the genomic DNA level (Supplemental Fig. 3a). The bone volume to tissue volume ratio (BV/TV) of *CD11b-Cre;ATF3*^*fl/fl*^ mice was indistinguishable from control mice in both femurs (Fig. 1b) and vertebrae (Fig. 1c and Supplemental Fig. 4a). Bone histomorphometric analyses revealed that the osteoclast surface/bone surface (Oc.S/BS), one of the indices of osteoclastic function, in addition to bone formation indices, such as bone formation rate (BFR) and osteoblast surface/bone surface (Ob.S/BS), were comparable between *CD11b-Cre;ATF3*^*fl/fl*^ mice and control mice (Fig. 1d, and Supplemental Fig. 4b,c). These results show that ATF3 deletion in osteoclast precursors does not affect bone volume and indices of osteoblastic and osteoclastic functions under the physiological condition.

We then investigated the possibility of the involvement of ATF3 expressed by osteoclast precursors in pathogenesis in an osteoporosis mouse model using GST-RANKL fusion protein injections. RANKL was injected in control and *CD11b-Cre;ATF3*^*fl/fl*^ mice at a daily rate over 2 days, followed by an analysis of bone phenotype. RANKL injection markedly induced bone loss in both femurs and vertebrae of control mice, whereas RANKL-induced bone loss was markedly impaired in *CD11b-Cre;ATF3*^*fl/fl*^ mice (Fig. 1e–h). Moreover, the RANKL-induced increase in osteoclastic parameters, Oc.S/BS, and the number of osteoclast/bone perimeter (N.Oc/B.Pm), were also repressed in *CD11b-Cre;ATF3*^*fl/fl*^ mice (Fig. 1i,j). Therefore, ATF3 could be a pivotal modulator of osteoclastogenesis and bone resorption *in vivo* under pathological conditions.

### No abnormalities are shown under the conditional deletion of ATF3 in osteoblasts under both physiological and pathological conditions

**Figure 2:**
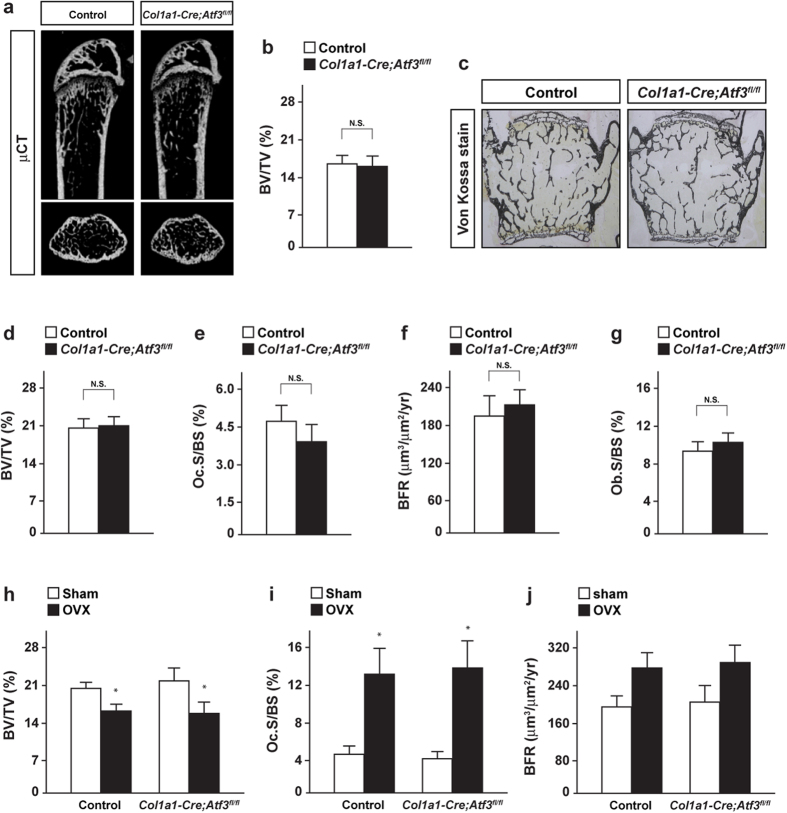
ATF3 expressed by osteoblasts is dispensable for bone formation and bone remodeling. (**a**) μCT analysis and (**b**) BV/TV of femurs, (**c**) Von Kossa staining, (**d**) BV/TV, (**e**) Oc.S/BS, (**f**) BFR and (**g**) Ob.S/BS of vertebrae of control and *Col1a1*-*Cre;ATF3*^*fl/fl*^ male mice at 12 week-old (control, *n* = 5; *Col1a1*-*Cre;ATF3*^*fl/fl*^, *n* = 6). (**h**) BV/TV, (**i**) Oc.S/BS and (**j**) BFR of ovariectomized mice (control-sham, *n* = 7; control-OVX, *n* = 8; *Col1a1*-*Cre;ATF3*^*fl/fl*^-Sham, *n* = 6; *Col1a1*-*Cre;ATF3*^*fl/fl*^-OVX, *n* = 7). *P < 0.05, significantly different from the value obtained for sham mice.

Further, we attempted to identify the importance of ATF3 expressed in osteoblasts on bone homeostasis *in vivo*. Osteoblast-specific ATF3 knockout mice were generated with *collagen type 1 alpha 1* (*Col1a1*)*-Cre* transgenic mice. Osteoblast-specific ATF3 knockout mice, hereafter referred to as *Col1a1-Cre;ATF3*^*fl/fl*^ mice, exhibited no apparent change in osteoblastic and osteoclastic parameters (Fig. 2e–g) as well as in the bone volume of both femurs and vertebrae (Fig. 2a–d). A marked Cre-mediated excision was confirmed in cultured osteoblasts of *Col1a1-Cre;Atf3*^*fl/fl*^ mice at the genomic DNA level (Supplemental Fig. 3b). To further ascertain the effect of ATF3 deletion in osteoblasts under the pathological condition, *Col1a1-Cre;ATF3*^*fl/fl*^ mice were subjected to ovariectomy (OVX), followed by bone analyses. OVX significantly induced bone loss in *Col1a1-Cre;ATF3*^*fl/fl*^ mice to a similar extent as in control mice (Fig. 2h). Moreover, both osteoclastic and osteoblastic parameters were altered by OVX to a similar extent in *Col1a1-Cre;ATF3*^*fl/fl*^ mice and control mice (Fig. 2i,j). These results indicated that ATF3 may not be implicated in osteoblastogenesis and bone homeostasis through its expression in osteoblasts *in vivo*.

### ATF3 deficiency abolishes RANKL-induced osteoclastogenesis *in vitro*

**Figure 3:**
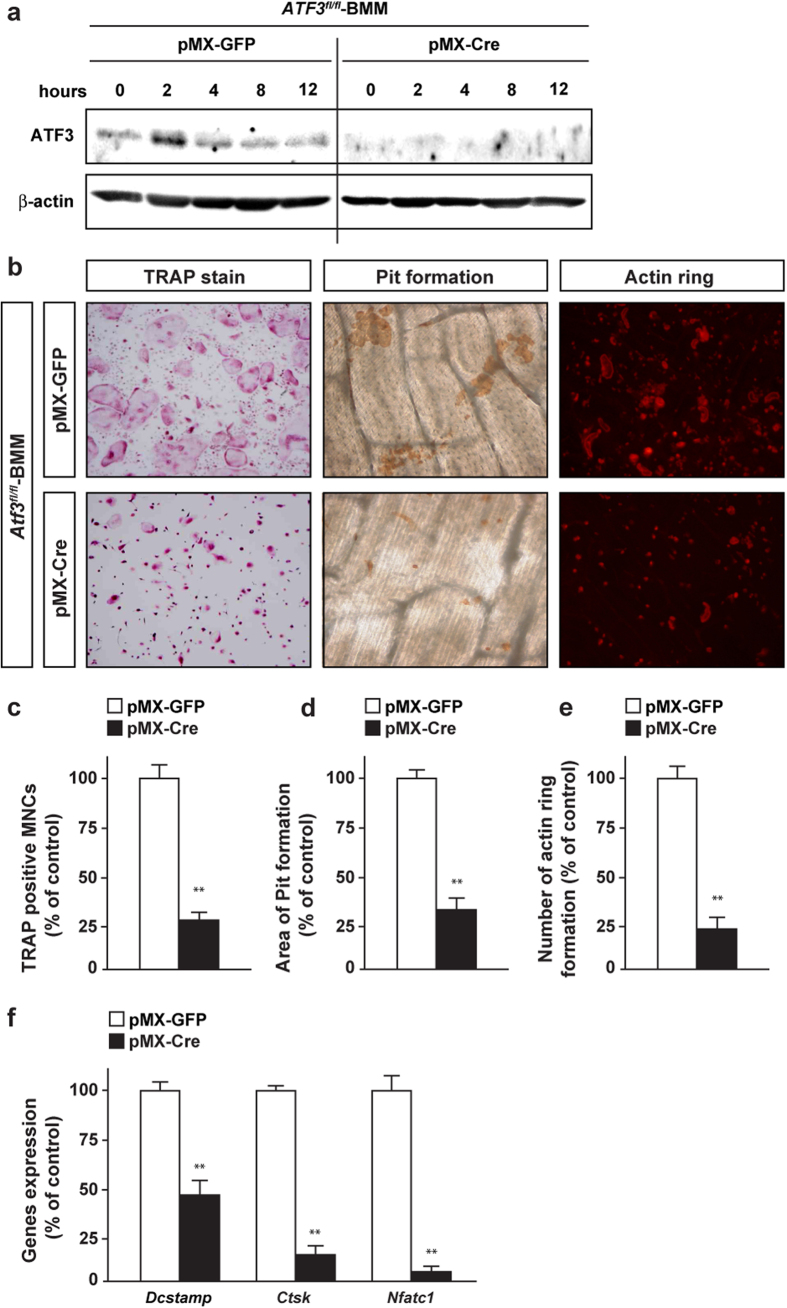
ATF3 deficiency represses osteoclastogenesis. BMM from *ATF3*^*fl/fl*^ mice was retrovirally infected with Cre recombinase, and subsequent stimulation with RANKL, followed by determination of (**a**) ATF3 expression, (**b,c**) TRAP stain, (**b,d**) Pit formation, (**b,e**) Actin ring, and (**f**) mRNA expression of osteoclast marker genes (*n* = 3–4). **P < 0.01, significantly different from the value obtained in cells infected with GFP.

We evaluated whether ATF3 regulates osteoclastogenesis through its expression in osteoclast lineages *in vitro. ATF3*^*fl/fl*^ mice-derived bone marrow macrophages (BMM) were infected with a retrovirus expressing Cre recombinase and subjected to differentiation by RANKL, followed by tartrate-resistant acid phosphatase (TRAP) staining, actin ring formation assay, and pit assay. ATF3 expression was markedly repressed in BMM infected with a retrovirus expressing Cre recombinase (Fig. 3a). The number of TRAP positive multinucleated cells, area of pit formation, and the number of actin ring formation, were significantly decreased in ATF3-deleted BMM (Fig. 3b–e). Moreover, the expression of the osteoclast differentiation and fusion markers, transmembrane 7 superfamily member 4 (*Dcstamp*), cathepsin K (*Ctsk*) and *Nfatc1* was markedly decreased in ATF3-deleted osteoclasts (Fig. 3f).

On the contrary, ATF3 deficiency did not alter the area of pit formation, when the same number of mature osteoclasts was used for pit formation assay (Supplemental Fig. 5). These results clearly show that ATF3 positively regulates osteoclast differentiation rather than maturation *in vitro*.

To reveal the role of ATF3 in osteoblasts on osteoclastogenesis, we performed co-culture experiments. *ATF3*^*fl/fl*^ mice-derived osteoblasts were retrovirally transfected with Cre recombinase and subsequent co-culture with WT mice-derived BMM, followed by TRAP staining. A similar number of TRAP positive cells were generated irrespective of *ATF3* deficiency in osteoblasts (Supplemental Fig. 6).

Jun dimerization protein 2 (Jdp2), which is the closest relative of ATF3 among AP-1 family members, reportedly plays an important role during osteoclastogenesis through modulating NFATc1 activity and inhibits ATF3 expression in fibroblasts and neutrophils^23^,^25^. To examine whether Jdp2 is implicated in *ATF3* expression during osteoclastogenesis, WT mice-derived BMMs were retrovirally transfected with sh-Jdp2 and subsequent RANKL stimulation, followed by determination of *ATF3* expression during osteoclastogenesis (at day 0–day 4). *ATF3* expression was significantly higher in Jdp2-knockdown BMM at day 0, 1, 3, and 4, indicating a contribution of Jdp2 in *ATF3* expression in osteoclasts (Supplemental Fig. 7).

### ATF3 deficiency represses RANKL-induced cell proliferation *in vitro* and *in vivo*

**Figure 4:**
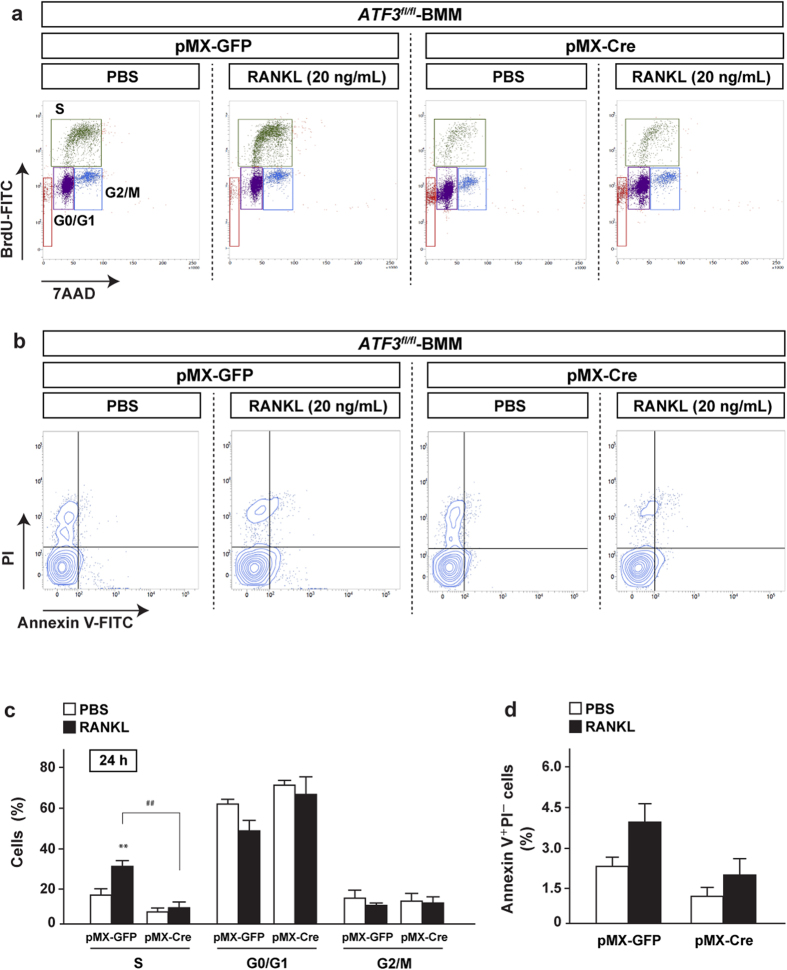
ATF3 deficiency blunts RANKL-induced cell proliferation *in vitro*. BMM from *ATF3*^*fl/fl*^ mice was retrovirally infected with Cre recombinase, and subsequent stimulation with RANKL, followed by treatment with BrdU. Cells were then analyzed for (**a,c**) BrdU incorporation assay or (**b,d**) cell death assay by flow cytometry (*n* = 5–8). **P < 0.01, significantly different from the value obtained in cells treated with PBS. ^##^P < 0.01, significantly different from the value obtained in RANKL-stimulated cells infected with GFP.

We focused on cell survival and proliferation of osteoclast precursors because of the observation that RANKL induced an increase in osteoclast number in control mice, and its increase was significantly repressed in *CD11b-Cre;ATF3*^*fl/fl*^ mice, as shown in Fig. 1j. In line with our observation *in vivo*, it has been reported that RANKL transiently increases cell proliferation of BMM prior to the triggering of the arrest of cell proliferation^26^. *ATF3*^*fl/fl*^ mice-derived BMM were infected with a retrovirus expressing Cre recombinase and subsequently stimulated with RANKL for the specified time periods, followed by determination of cell proliferation and cell survivability. Flow cytometric measurements after BrdU incorporation revealed that RANKL stimulation for 24 h (but not for 8 h) significantly increased the number of cells in the S-phase of control cells (Fig. 4a,c and Supplemental Fig. 8a). In contrast, in ATF3-deleted BMM, the RANKL-induced increase in the number of cells in the S-phase was markedly reduced (Fig. 4a,c). The number of cells in the S-phase tended to decrease in ATF3-deleted BMM with PBS treatment, indicating that ATF3 may be involved in basal cell proliferation in addition to the presence of RANKL signaling. On the contrary, ATF3 deficiency did not affect the number of apoptotic cells in BMM irrespective of the presence or absence of RANKL (Fig. 4b,d).

**Figure 5:**
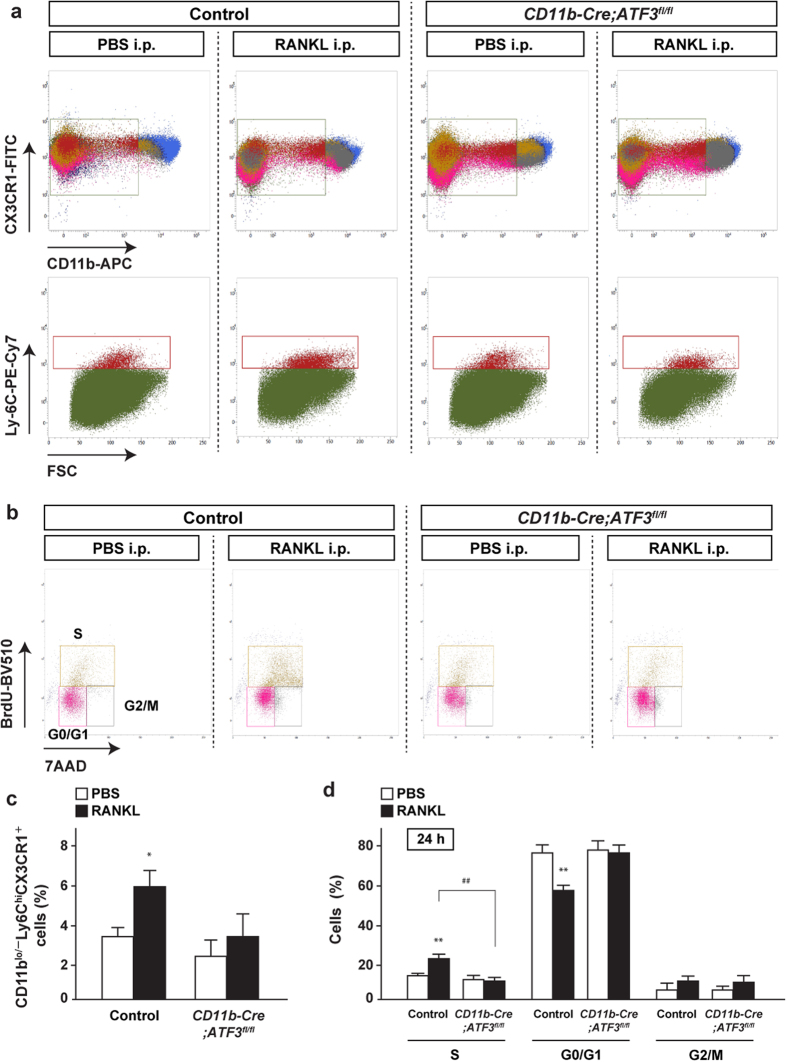
ATF3 deficiency blunts RANKL-induced cell proliferation *in vivo*. Control mice and *CD11b*-*Cre;ATF3*^*fl/fl*^ male mice were i.p. injected with RANKL, and subsequent treatment with BrdU (control-PBS, *n* = 5; control-RANKL, *n* = 6; *CD11b*-*Cre;ATF3*^*fl/fl*^-PBS, *n* = 5; *CD11b*-*Cre;ATF3*^*fl/fl*^-RANKL, *n* = 7). Bone marrow cells were isolated 24 h after RANKL administration and then analyzed for (**a,c**) the ratio of CD11b^lo/−^Ly6C^hi^CX3CR1^+^ cells and (**b,d**) BrdU incorporation in CD11b^lo/−^Ly6C^hi^CX3CR1^+^ cells by flow cytometry. *P < 0.05, **P < 0.01, significantly different from the value obtained in mice treated with PBS. ^##^P < 0.01, significantly different from the value obtained in RANKL-injected control mice.

To determine whether this was true *in vivo*, GST-RANKL fusion protein was administered to control and *CD11b-Cre;ATF3*^*fl/fl*^ mice, followed by determination of cell proliferation. To more precisely determine cell proliferation of osteoclast precursors *in vivo*, the number of cells in the S-phase was analyzed in CD11b^lo/−^Ly6C^hi^CX3CR1^+^ cells because it has been previously shown that CD11b^lo/−^Ly6C^hi^CX3CR1^+^ cells in the bone marrow contain the majority of osteoclast precursor activity^27^. The ratio of CD11b^lo/−^Ly6C^hi^CX3CR1^+^ cells in bone marrow cells was significantly increased in control mice, but not in *CD11b-Cre*;*ATF3*^*fl/fl*^ mice 24 h after RANKL injection (Fig. 5a,c). The S-phase CD11b^lo/−^Ly6C^hi^CX3CR1^+^ cells of control mice were significantly increased 24 h (but not 12 h) after RANKL administration, but its increase by RANKL was markedly impaired in *CD11b-Cre;ATF3*^*fl/fl*^ mice-derived cells (Fig. 5b,d and Supplemental Fig. 8b). Moreover, cell proliferation of osteoclast precursors was determined in cfms^+^ cells, which are known to have high capacity for differentiating into multinuclear osteoclasts^28^. As shown in CD11b^lo/−^Ly6C^hi^CX3CR1^+^ cells, RANKL-induced increase in the S-phase CD11b^+^cfms^+^ cells of control mice was markedly impaired in *CD11b-Cre;ATF3*^*fl/fl*^ mice-derived cells (Supplemental Fig. 9). Collectively, these findings indicate that ATF3 deficiency attenuates RANKL-induced proliferation of osteoclast precursor both *in vitro* and *in vivo*.

### Cyclin D1 is implicated in the ATF3 deficiency-induced repression of osteoclast differentiation

**Figure 6:**
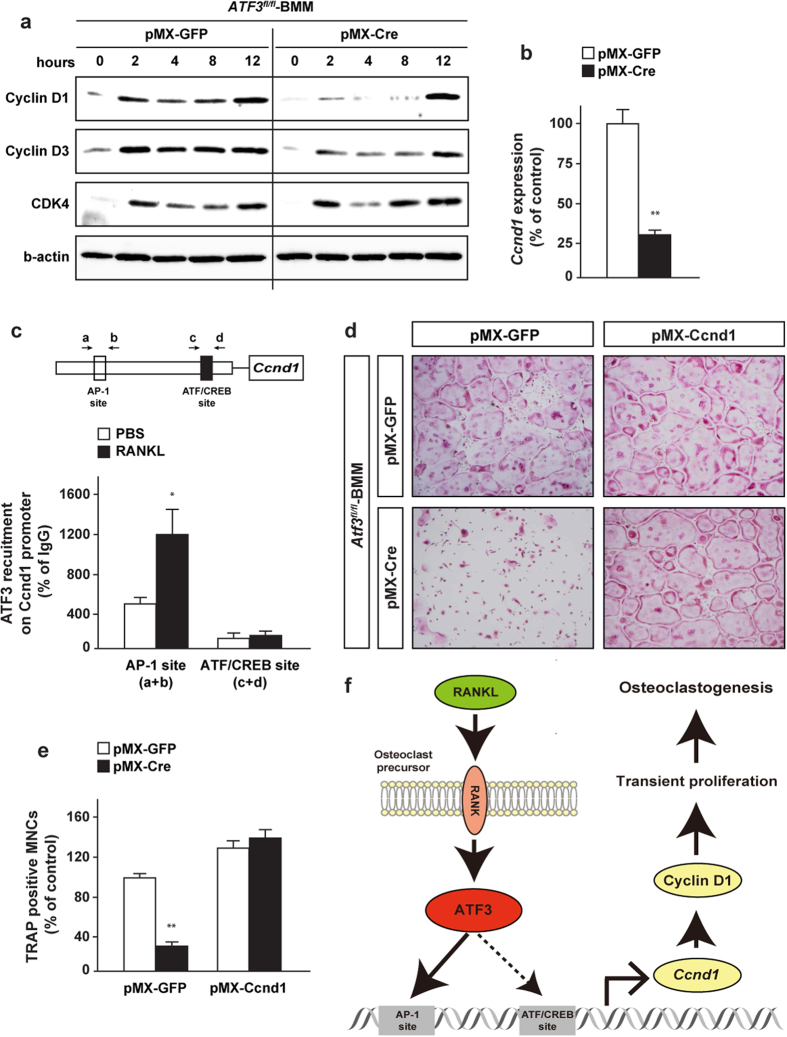
ATF3 positively regulates *Ccnd1* expression in osteoclast precursors. BMM from *ATF3*^*fl/fl*^ mice was retrovirally infected with Cre recombinase, and subsequent treatment with RANKL, followed by determination of (**a**) Cyclin-related proteins expression, (**b**) *Ccnd1* expression (*n* = 4). (**c**) BMM from WT mice were treated with RANKL and examined by ChIP assay using anti-ATF3 antibody (*n* = 3). (**d,e**) BMM from *ATF3*^*fl/fl*^ mice was retrovirally infected with Cre recombinase and CyclinD1, and subsequent treatment with RANKL, followed by determination of TRAP stain (*n* = 4). (**f**) Schematic model of this study. *P < 0.05, **P < 0.01, significantly different from control values obtained in cells (**c**) treated with PBS or (**b,e**) infected with GFP.

To elucidate the mechanism of ATF3 regulation of RANKL-induced proliferation of osteoclast precursors, the expression pattern of cell proliferation-related protein was determined in ATF3-deficient cells. Among these, cyclin D1 expression was markedly decreased in ATF3-deficient BMM, in addition to cyclin D3 to a lesser extent (Fig. 6a). Moreover, cyclin D1 mRNA expression was significantly decreased in ATF3-deleted BMM (Fig. 6b). Although an ATF/CREB binding site was previously identified in the cyclin D1 promoter^29^ (Fig. 6c), the binding of ATF3 to the site was detected to a lesser extent in either the presence or absence of RANKL in BMM (Fig. 6c). It has been previously reported that ATF3 binds to the AP-1 responsive site of cyclin D1 promoter, leading to its transcriptional activation in hepatocytes^30^. Therefore, we examined whether ATF3 binds to its site in BMM. The recruitment of ATF3 to the AP-1 responsive site of cyclin D1 promoter was detected in BMM under the basal condition, and it was significantly increased by RANKL (Fig. 6c).

Finally, we determined the possibility of the implication of cyclin D1 in the repression of cell differentiation observed in ATF3-deficient BMM. *ATF3*^*fl/fl*^ mice-derived BMM were infected with a retrovirus expressing Cre recombinase and cyclin D1 and subsequently stimulated with RANKL, followed by determination of TRAP positive multinucleated cells. An impairment of osteoclastogenesis was almost completely rescued in retroviral transduction of cyclin D1 into ATF3-deleted BMM (Fig. 6d,e). These results suggest that ATF3 regulates osteoclast differentiation through the modulation of cyclin D1-dependent cell proliferation (Fig. 6f).

## Discussion

The essential relevance of the present findings is that the transcription factor ATF3 regulates bone resorption and bone remodeling under the pathological condition through its expression in osteoclast precursors. ATF3 has been demonstrated to be involved in the pathogenesis of various diseases^16^,^18^,^19^,^31^. The present study indicates the possibility of the implication of ATF3 in metabolic bone diseases. ATF3 deficiency in osteoblasts did not alter bone volume and bone formation parameters *in vivo* under both pathological and physiological conditions. Although further *in vivo* examination should be conducted to completely exclude the possible involvement of ATF3 in bone formation and bone remodeling through its expression in osteoblasts, ATF3 would be a pivotal and specific transcription factor capable of regulating bone resorption and bone remodeling under the pathological condition through its expression in osteoclast lineages.

In the present study, *CD11b-Cre;ATF3*^*fl/fl*^ mice showed protection against bone loss and an increase in the number of osteoclasts induced by RANKL administration. The RANKL-induced increase in cell proliferation was abolished in ATF3-deficient osteoclast precursors, both *in vitro* (BMM) and *in vivo* (CD11b^lo/−^Ly6C^hi^CX3CR1^+^ cells and CD11b^+^cfms^+^ cells in bone marrow cells), along with repression of cyclin D1 expression in BMM. Moreover, the introduction of cyclin D1 into ATF3-deficient osteoclast precursors almost completely rescued the impairment of osteoclastogenesis. Although further study should be conducted to determine whether osteoclast-specific deletion of cyclin D1 in mice could display protection from RANKL-induced bone loss, the mechanism underlying the regulation by ATF3 of osteoclastogenesis and bone resorption appears to at least in part involve cyclin D1-dependent proliferation of osteoclast precursors. It was reported that RANKL-induced osteoclastogenesis is regulated by cyclin-dependent protein kinases (CDKs) and cyclin-dependent kinase inhibitors (CKIs) such as Cdk6 or p21/p27^32^,^33^. Similarly, in the present study, we could not exclude the possibility that CDKs and CKIs, as well as another cyclin members, could contribute to the regulation of ATF3-dependent cell proliferation of osteoclast precursors and osteoclastogenesis.

ATF3 has been shown to repress or activate the transcription of target genes, depending on various factors including cell type, binding sequences, or complex partners. Indeed, ATF3 has been shown to repress cyclin D1 expression, possibly through its binding to the ATF/CREB binding site in chondrocytes, whereas it binds to the cyclin D1 AP-1 binding site, leading to its transcriptional activation in hepatocytes^30^,^34^. Moreover, a c-Fos/c-Jun complex activates cyclin D1 promoter activity by binding to the AP-1 binding site in response to growth factor signaling^35^,^36^, indicating the possible physical interaction between c-Fos/c-Jun and ATF3 on the AP-1 binding site of cyclin D1 promoter. Although the exact mechanism underlying the regulation by ATF3 of Cyclin D1 transcription remained unclear, the present study showed that ATF3 transcriptionally regulated cyclin D1 expression though its binding to the AP-1 binding site rather than ATF/CREB binding site in osteoclast precursors.

In conclusion, the results of the present study support the assertion that the transcription factor ATF3 is functionally expressed by osteoclast precursors to promote cell proliferation required for osteoclastogenesis during bone resorption and remodeling under the pathological condition. The novel player ATF3 and RANK-ATF3-cyclin D1 axis could thus be a future target for the discovery and development of a drug useful for the treatment and therapy of a variety of metabolic bone diseases relevant to abnormal proliferation of osteoclast precursors.

## Materials and Methods

### Materials

We are very grateful to Dr. S.L. Teitelbaum (Washington University, St. Louis, MO, USA), and T. Kitamura (Tokyo University, Tokyo, Japan) for generously providing GST-RANKL vector and PLAT-E cells, respectively. Recombinant mouse RANKL was purchased from R&D Systems International (Minneapolis, MN, USA). Antibodies were from the following companies: anti-ATF3 and anti-β-Actin were from Santa Cruz Biotechnology (Santa Cruz, CA, USA); anti-Cyclin D1, anti-Cyclin D3 and anti-CDK4 were from Cell Signaling Technology (Danvers, MA, USA). THUNDERBIRD SYBR qPCR Mix was supplied by TOYOBO (Osaka, Japan). Other chemicals used were all of the highest purity commercially available.

### Mice

The protocol used here meets the guideline of the Japanese Society for Pharmacology and was approved by the Committee for Ethical Use of Experimental Animals at Kanazawa University. Mice harboring the floxed ATF3 alleles (*ATF3*^*fl/fl*^)^37^ were crossed with *CD11b-Cre* mice^38^ to generate *CD11b-Cre;ATF3*^*fl/*+^ mice. Because *CD11b-Cre* is on the Y chromosome, male *CD11b-Cre;ATF3*^*fl/*+^ mice were crossed with female *ATF3*^*fl/*+^ mice to obtain *CD11b-Cre;ATF3*^+/+^ and *CD11b-Cre;ATF3*^*fl/fl*^ male mice. *ATF3*^*fl/fl*^ mice were crossed with *Col1a1-Cre* to generate *Col1a1-Cre;ATF3*^*fl/*+^ mice, and their progeny intercrossed to obtain *Col1a1-Cre;ATF3*^*fl/fl*^ mice. These mutant mice were backcrossed more than five generations with C57BL/6J. Mice were bred under standard animal housing conditions at 23 ± 1 °C with humidity of 55% and a light/dark cycle of 12 h, with free access to food and water. Genotyping was performed by PCR using tail genomic DNA. The numbers of animals used per experiment are stated in the figure legends.

### Cell sorting

Total bone marrow cells were obtained from femur and tibia, followed by depletion of red blood cells by soaking in 0.15 M NH_4_Cl for 5 min. Cells were then resuspended in PBS containing 2% FBS and incubated for 30 min at 4 degree with Allophycocyanin (APC) conjugated anti-CD11b antibody (M1/70) (BioLegend) and 7-Amino-ActinomycinD (7-AAD) for dead cell exclusion. Immunostained cells were washed and analyzed on FACS AriaII cell sorter (BD Biosciences), and APC positive cells were sorted.

### Cell cycle and cell death assay

Bone marrow cells were harvested 1 h after intraperitoneal injection of BrdU (100 mg/kg), and subsequent incubation with APC-anti-CD11b (M1/70), FITC-anti-CX3CR1 (SA011F11), BV421-anti-cfms (AFS98) antibodies (BioLegend), and PE-Cy7-anti-Ly-6C (AL-21), BV510-anti-BrdU (3D4) antibodies (BD Biosciences). Immunostained cells were prepared for analysis of BrdU incorporation using the FITC BrdU Flow Kit (BD Biosciences) by flow cytometric analysis using the FACS Verse (BD Biosciences). Data were analyzed by FACS Suite software (BD Biosciences). For analysis of the cell cycle of primary osteoclasts *in vitro*, cells were cultured with 10 μM BrdU for 45 min, and prepared for analysis of BrdU incorporation using the FITC BrdU Flow Kit. Cell death assay was performed with FITC-Annexin V (BD Biosciences) and Propidium Iodide (PI) by FACS verse.

### Operation of OVX and RANKL administration

Eight week-old mice were anesthetized by an intraperitoneal injection of pentobarbital and subjected to OVX or sham operation under aseptic environments as described previously^39^. Mice were killed by decapitation 28 days after operation.

Eight week-old mice were intraperitoneally administrated with GST-RANKL fusion protein at a rate of 2 mg/kg daily for 2 days. Mice were killed by decapitation 12 h after final injection.

### Bone histomorphometric analyses

Bone histomorphometric analyses were performed on vertebrae as previously described^40^. Briefly, vertebrae were fixed with 10% formalin, followed by dehydration in different concentrations of ethanol and subsequent embedding in methyl methacrylate resin according to standard protocols. BV/TV ratio was measured by Von Kossa staining. BFR was analyzed by the calcein double-labeling method. Calcein was injected to mice twice with an interval of 3 days, and then mice were killed 2 days after the last injection. Osteoblast and osteoclast parameters were analyzed by staining with toluidine blue or with TRAP, respectively. Analyses were performed using the Osteomeasure Analysis System (Osteometrics) according to standard protocols. Two-dimensional images of the distal femurs were obtained by μCT scanning using a Scan Xmate-L090 (Comscan Tecno) with high resolution of 12 μm/pixel, voltage of 75 kV, and current of 100 μA, as described previously^41^.

### Retroviral transfection

Retroviral vectors were transfected into PLAT-E cells using the calcium carbonate method. Virus supernatants were collected 48 h after transfection, and then cells were infected with virus supernatants for 72 h in the presence of 4 μg/ml polybrene. Cells were then subjected to selection by culture with 1 μg/ml puromycin for 3 days before usage for experiments. The oligonucleotides for Jdp2 shRNA (5′-GATCCGTTTCTGCAGAGGGAGTCAGACTCGAGTCTGACTCCCTCTGCAGAAACTTTTTG-3′ and 5′-GATCCGTTTCTGCAGAGGGAGTCAGACTCGAGTCTGACTCCCTCTGCAGAAACTTTTTG-3′) were synthesized, annealed and inserted into the RNAi-Ready pSIREN-RetroQ vector.

### Co-culture experiment

Bone marrow cells obtained from the long bones were stimulated with 100 ng/ml macrophage colony-stimulating factor (M-CSF) for 3 days; we used these cells as BMM. Calvarial osteoblasts were co-cultured with BMM in the presence of 1,25-dihydroxycholecalciferol (vitamin D_3_) at 10 nM for 8 days, followed by TRAP staining to count the number of TRAP-positive multinucleated cells.

### Culture of osteoclasts and TRAP staining, the actin ring assay, and the pit formation assay

BMM were seeded on 48 well-plates (Nunc) at a density of 2.5 × 10^4 ^cells/well and were cultured in the presence of 20 ng/ml M-CSF and 20 ng/ml RANKL for 4 days consecutively. TRAP staining, the actin ring assay, and the pit formation assay were performed as previously described^42^.

### Isolation of osteoclasts

Isolation of osteoclasts was performed from long bones as previously described^43^. In brief, the long bones were minced into small pieces, and cells were dissociated from bone fragments by vigorous vortex in αMEM. After removal of bone fragments by sedimentation under normal gravity, the unfractionated bone cells were seeded on collagen gel and were incubated in αMEM with 5% FBS for 4 h. The cells were sequentially treated with 0.001% Pronase E and 0.02% EDTA for 5 min, 0.01% collagenase for 5 min and 0.1% collagenase for 10 min. Cells released by final treatment were collected and cultured as osteoclasts.

### Real-time based quantitative PCR

Total RNA was extracted from cells, followed by synthesis of cDNA with reverse transcriptase and oligo-dT primer. The cDNA samples were then used as templates for real-time PCR analysis, which was performed on an MX3005P instrument (Agilent Technologies), by using specific primers for each gene (Supplemental Table 1). Expression levels of the genes examined were normalized by using the 36b4 expression levels as an internal control for each sample.

### Immunoblotting analysis

Cultured cells were solubilized in lysis buffer (10 mM Tris-HCl, 150 mM NaCl, 0.5 mM EDTA, 10 mM NaF, 1% Nonidet P-40, pH 7.4) containing protease inhibitor cocktail (1 mM APMSF, 1 μg/ml Leupeptin 1 μg/ml PepstatinA 1 μg/ml Antipain). Samples were then subjected to SDS-PAGE, followed by transfer to PVDF membranes and subsequent immunoblotting assay. Quantification was performed by densitometry using ImageJ.

### ChIP assay

ChIP experiments were performed following the protocol provided with the ChIP assay kit (Upstate Biotechnology) as described previously^44^. Cells were treated with formaldehyde for crosslinking and subsequently subjected to sonication in lysis buffer (50 mM Tris-HCl, 10 mM EDTA, 1% SDS, pH 8.0) containing protease inhibitor cocktail. Immunoprecipitation was performed with the anti-ATF3 antibody, followed by extraction of DNA with phenol/chloroform and subsequent PCR with specific primers (Supplemental Table 2).

### Data analysis

Results are all expressed as the mean ± S.E. and the statistical significance was determined by the two-tailed and unpaired Students’ *t*-test or the one-way analysis of variance ANOVA with Bonferroni/Dunnett post hoc test.

## Additional Information

**How to cite this article**: Fukasawa, K. *et al.* ATF3 controls proliferation of osteoclast precursor and bone remodeling. *Sci. Rep.* **6**, 30918; doi: 10.1038/srep30918 (2016).

## Supplementary Material

Supplementary Information
