# Supplementary material for: ATF3 controls proliferation of osteoclast precursor and bone remodeling
Source: Sci Rep. 2016 Aug 2;6:30918. doi: 10.1038/srep30918 (PMC4969588; doi:10.1038/srep30918)

ATF3 controls proliferation of osteoclast precursor and bone remodeling

Kazuya Fukasawa, Gyujin Park, Takashi Iezaki, Tetsuhiro Horie, Takashi Kanayama, Kakeru Ozaki, Yuki Onishi, Yoshifumi Takahata, Yukio Yoneda, Takeshi Takarada, Shigetaka Kitajima, Jean Vacher and Eiichi Hinoi.

Supplemental Table 1. List of primers used for real-time PCR.

Genes	Upstream (5'-3')	Downstream (5'-3')
Ccnd1	TGGATGCTGGAGGTCTGTGAG	AGAGGCCACGAACATGCAG
ATF3	GAGGATTTTGCTAACCTGACACC	TTGACGGTAACTGACTCCAGC
Dcstamp	GACCTTGGGCACCAGTATTT	CAAAGCAACAGACTCCCAAA
Ctsk	GAAGAAGACTCACCAGAAGCAG	TCCAGGTTATGGGCAGAGATT
Nfatc1	CCCGTTGCTTCCAGAAAATA	CCCGTTGCTTCCAGAAAATA

Supplemental Table 2. List of primers used for ChIP assay.

Fragments	Upstream (5'-3')	Downstream (5'-3')
Ccnd1 promoter **AP-1 site**	GGAGAAACACCACCACCCT CAAC	TATTAGTCGCCCTTCCAGGAA CCAG
Ccnd1 promoter CREB/ATF **site**	TGCATATCTACGAAGGCTGA GG	CAGAGATCAAAGCCGGGCAG

Supplemental Figure legends

Supplemental Figure 1. Gene expression profile in osteoclasts *in vitro*. BMM from WT mice were stimulated with RANKL, followed by determination of mRNA expression of ATF family at day 0 to day 4 (n = 4).

Supplemental Figure 2. Gene expression profile in osteoblasts *in vitro*. Calvarial osteoblasts from WT mice were stimulated with ascorbic acid and β-glycerophosphate, followed by determination of mRNA expression of ATF family at day 0 to day 28 (n = 4).

Supplemental Figure 3. Genotyping of DNA isolated from BMM and osteoblasts. Genomic DNA was isolated from (a) CD11b positive cells from *CD11b-Cre;ATF3*$^{fl/fl}$ mice, and (b) osteoblasts from *Col1a1-Cre;ATF3*$^{fl/fl}$ mice, and subsequent determination of floxed allele and deletion allele by PCR.

Supplemental Figure 4. Phenotype of *CD11b-Cre;ATF3*$^{fl/fl}$ mice. (a) Von Kossa staining, (b) BFR and (c) Ob.S/BS of vertebrae of control and *CD11b-Cre;ATF3*$^{fl/fl}$ male mice at 12 week-old (control, n = 7; *CD11b-Cre;ATF3*$^{fl/fl}$, n = 9).

Supplemental Figure 5. Pit formation assay. Osteoclasts prepared from *ATF3*$^{fl/fl}$ mice were retrovirally infected with Cre recombinase, and subsequent pit formation assay (n = 3).

Supplemental Figure 6. Co-culture experiment. Calvarial osteoblasts from *ATF3*$^{fl/fl}$ mice was retrovirally infected with Cre recombinase, and subsequent co-culture with WT mice-derived BMM, followed by TRAP staining (n = 3).

Supplemental Figure 7. Involvement of Jdp2 in *ATF3* expression in osteoclasts. WT mice-derived BMMs were retrovirally transfected with sh-Jdp2, and subsequent RANKL stimulation, followed by determination of *ATF3* expression during osteoclast differentiation (n = 4).

Supplemental Figure 8. Time course of cell proliferation assay *in vitro* and *in vivo*. BrdU incorporation was determined in (a) BMM stimulated with RANKL for 8 h, and (b) $CD11b^{lo/-}Ly6C^{hi}CX3CR1^{+}$ cells of control mice 12 h after RANKL administration (n = 3).

Supplemental Figure 9. Cell proliferation assay in $CD11b^{+}cfms^{+}$ cells. Control mice and *CD11b-Cre;*$ATF3^{fl/fl}$ male mice were i.p. injected with RANKL, and subsequent treatment with BrdU (control-PBS, n = 5; control-RANKL, n = 6; *CD11b-Cre;*$ATF3^{fl/fl}$-PBS, n = 5; *CD11b-Cre;*$ATF3^{fl/fl}$-RANKL, n = 7). Bone marrow cells were isolated 24 h after RANKL administration and then analyzed for (a and c) the ratio of $CD11b^{+}cfms^{+}$ cells and (b and d) BrdU incorporation in $CD11b^{+}cfms^{+}$ cells by flow cytometry. *P < 0.05, **P < 0.01, significantly different from the value obtained in mice treated with PBS. $^{\#\#}$P<0.01, significantly different from the value obtained in RANKL-injected control mice.

Supplemental Figure 1

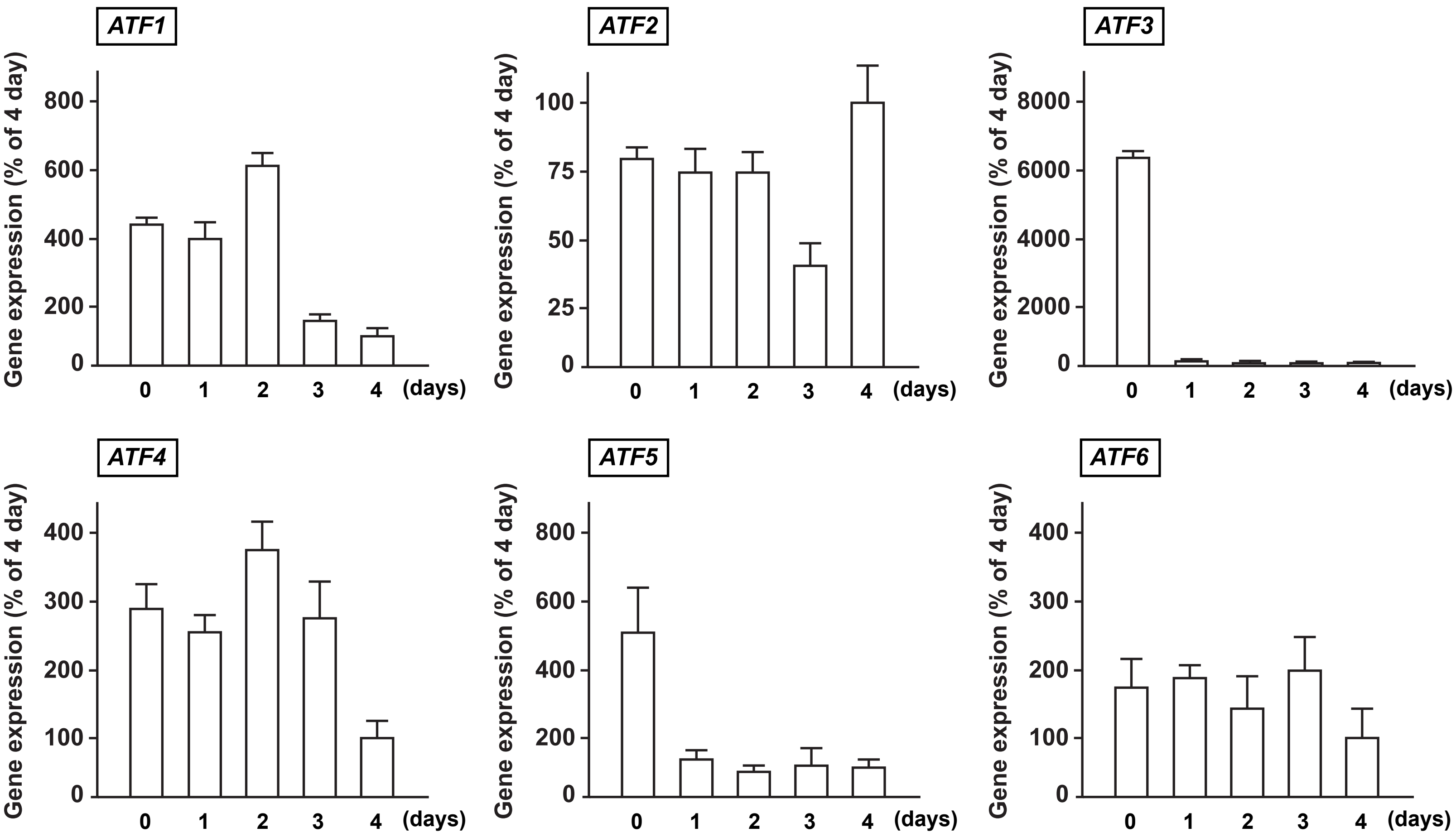

Supplemental Figure 2

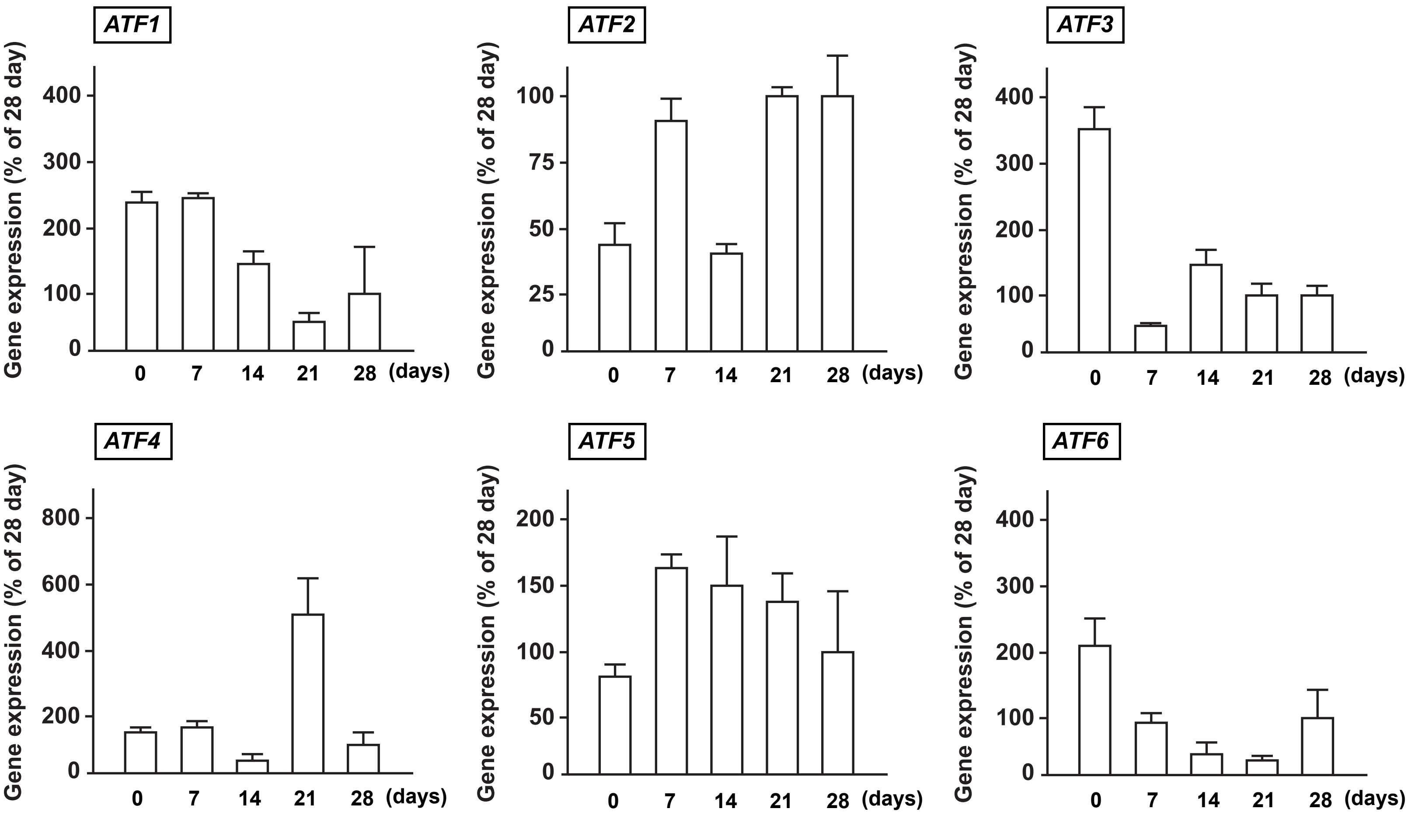

Supplemental Figure 3

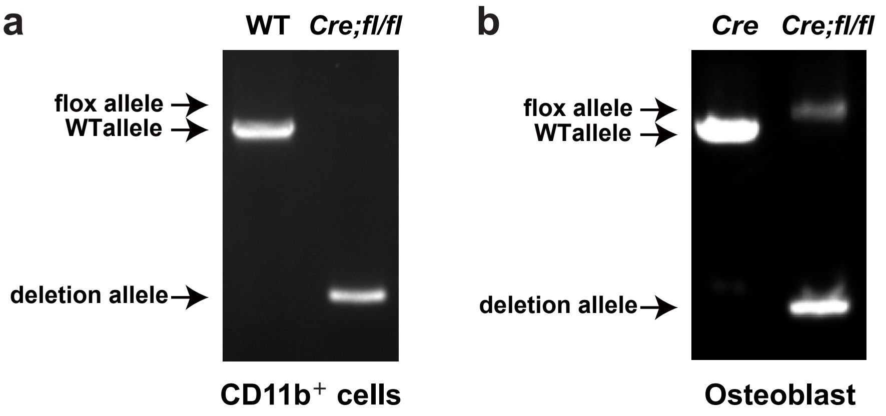

Supplemental Figure 4

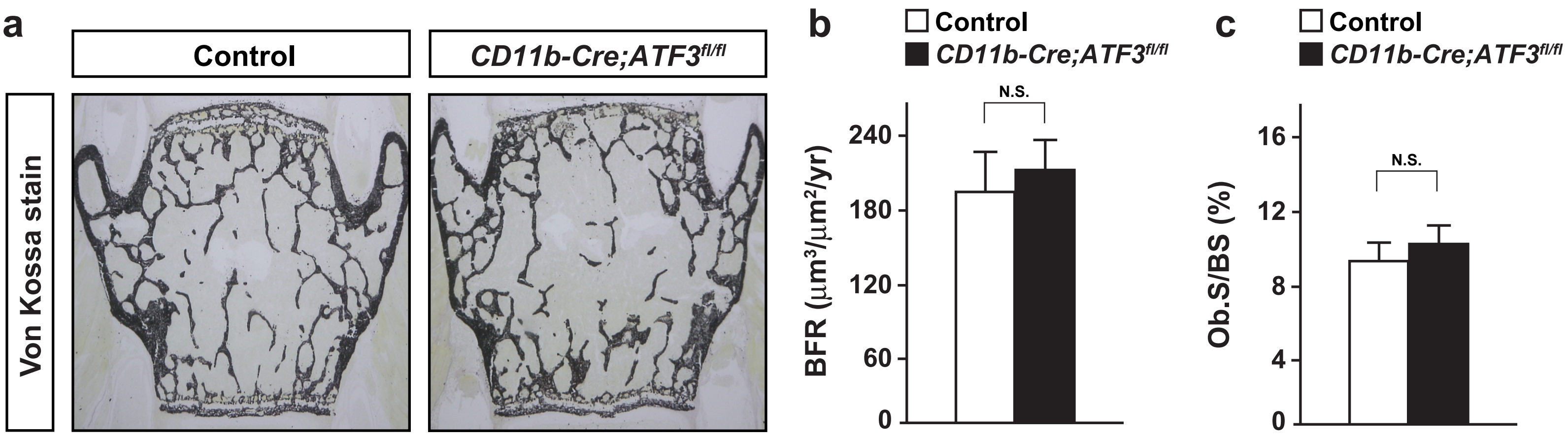

Supplemental Figure 5

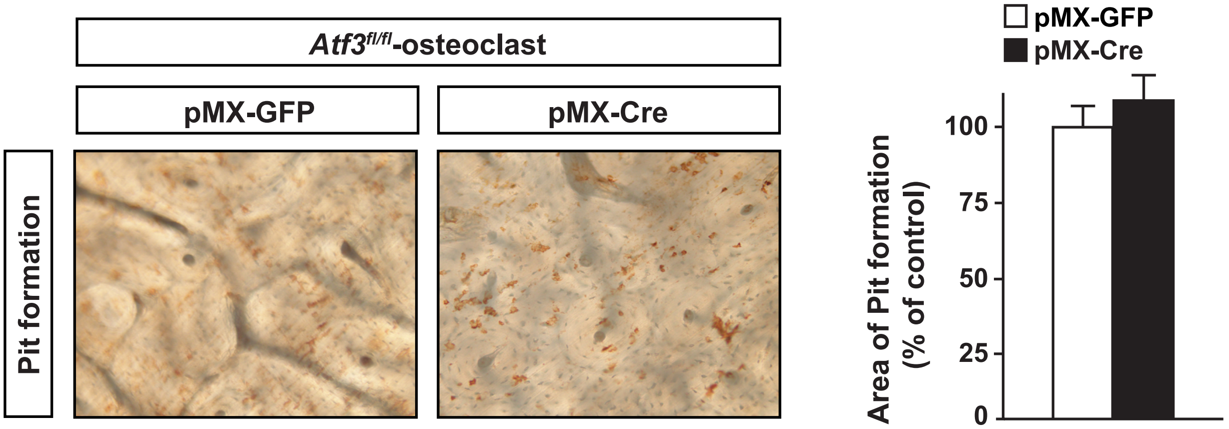

Supplemental Figure 6

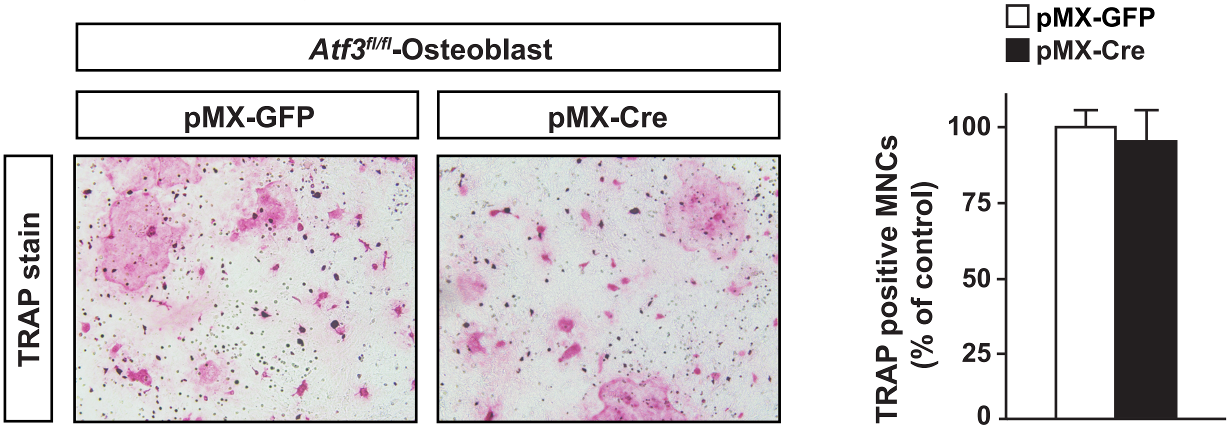

Supplemental Figure 7

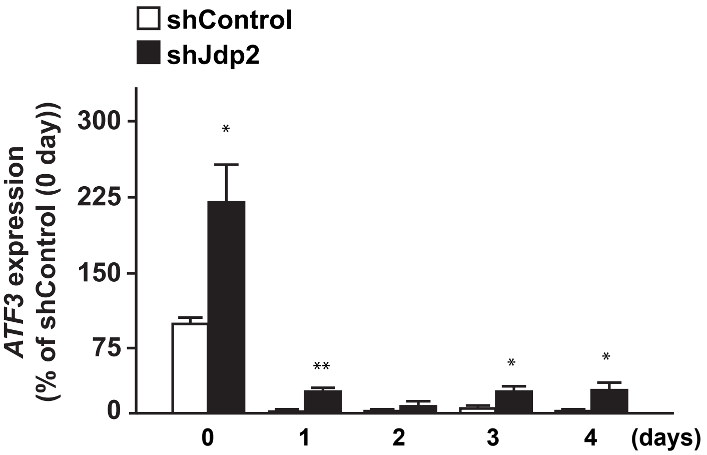

Supplemental Figure 8

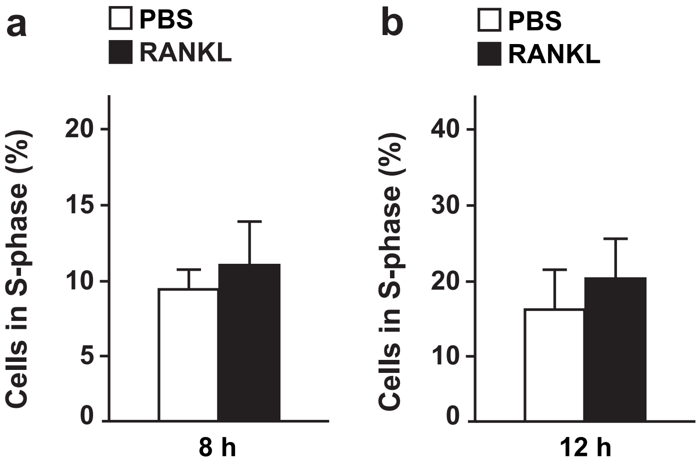

Supplemental Figure 9

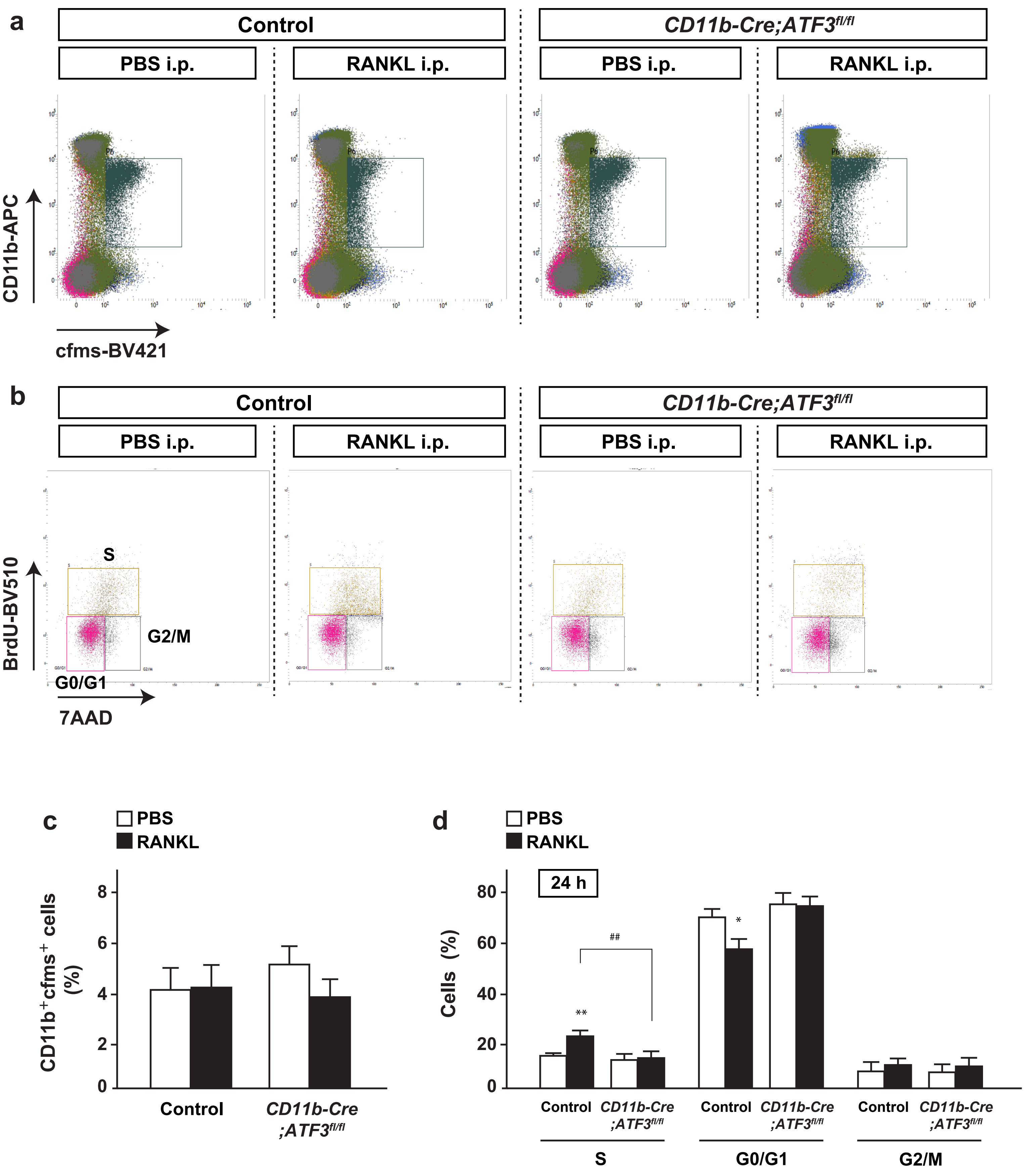

Supplement: Supplementary Information [file srep30918-s1.pdf]
